# Prognostic Value of 72-h Reassessment Using SOFA Score, C-Reactive Protein, and Lactate in a 72-h Landmark Cohort of Intensive Care Patients with Gram-Negative Sepsis

**DOI:** 10.3390/medicina62081531

**Published:** 2026-08-09

**Authors:** Mustafa Uğuz, Berfin Çirkin Doruk

**Affiliations:** Department of Infectious Diseases, Mersin City Training and Research Hospital, 33240 Mersin, Türkiye; berfin.cirkindoruk@hotmail.com

**Keywords:** gram-negative bloodstream infection, sepsis, intensive care unit, SOFA score, C-reactive protein, lactate, 28-day mortality, 72 h landmark cohort

## Abstract

*Background and Objectives*: Sepsis caused by Gram-negative bloodstream infections is associated with substantial mortality in intensive care units (ICUs). Admission measurements reflect initial disease severity but may not adequately capture subsequent clinical evolution or treatment response. This study evaluated whether a model based on 72 h reassessment demonstrated greater discrimination than a baseline model for mortality occurring between the 72 h landmark and day 28. *Materials and Methods*: This retrospective, single-center cohort study included adult ICU patients with Sepsis-3-defined sepsis and confirmed Gram-negative bloodstream infection admitted between January 2018 and November 2023. The primary analysis was conducted as a 72 h landmark cohort analysis with a fixed-horizon binary outcome and was restricted to patients who were alive and under observation at 72 h and had complete clinical and laboratory data. Separate multivariable logistic regression models were constructed using baseline and 72 h variables. Model discrimination was assessed using the area under the receiver operating characteristic curve (AUC), and the AUCs were compared using DeLong’s test for correlated ROC curves. Internal validation of the final 72 h model was performed using 1000 bootstrap resamples. *Results*: A total of 422 patients were included, of whom 134 (31.8%) died within 28 days. Baseline septic shock (adjusted odds ratio [OR] = 3.994; 95% confidence interval [CI]: 2.296–6.949; *p* < 0.001), higher 72 h SOFA score (adjusted OR = 1.603 per point; 95% CI: 1.424–1.805; *p* < 0.001), higher 72 h C-reactive protein level (adjusted OR = 1.005 per mg/L; 95% CI: 1.001–1.008; *p* = 0.005), and higher 72 h lactate level (adjusted OR = 1.189 per mmol/L; 95% CI: 1.007–1.403; *p* = 0.041) were independently associated with mortality occurring between the 72 h landmark and day 28. The 72 h model demonstrated greater discrimination than the baseline model (AUC: 0.867 vs. 0.799; AUC difference = 0.068; 95% CI: 0.027–0.109; *p* = 0.0012). The optimism-corrected AUC after bootstrap validation was 0.861. *Conclusions*: Among patients with Gram-negative sepsis who were alive at the 72 h landmark, a model incorporating baseline septic shock, 72 h SOFA score, CRP, and lactate demonstrated greater discrimination than the baseline model for subsequent mortality through day 28. These findings support the clinical relevance of 72 h reassessment for risk stratification, although external validation is required before routine clinical implementation.

## 1. Introduction

Sepsis remains one of the most critical syndromes encountered in intensive care units (ICUs) and continues to be associated with substantial mortality and morbidity. Global estimates indicate that sepsis contributes to nearly one-fifth of all deaths worldwide, highlighting its persistent importance as a major public health problem [1]. Timely initiation of appropriate antimicrobial therapy and organ support depends on the early identification of patients at high risk of deterioration [2]. Accordingly, the identification of reliable and clinically accessible prognostic markers remains an important component of sepsis management.

C-reactive protein (CRP) and procalcitonin (PCT) are widely used in the diagnosis and follow-up of sepsis and in the assessment of treatment response [3,4,5]. Increasing attention has been directed not only toward single measurements but also toward changes in these biomarkers over time. Previous studies have shown that an insufficient reduction in CRP during the first 72 h or failure of PCT concentrations to decline as expected may be associated with increased mortality [6,7]. Similarly, elevated lactate levels and inadequate lactate clearance during serial measurements in the first 48–72 h have been linked to poor outcomes, reflecting persistent tissue hypoperfusion and metabolic dysfunction [8]. However, evidence specifically evaluating the prognostic contribution of routine 72 h reassessment in patients with Gram-negative bloodstream infections remains limited.

Gram-negative bloodstream infections represent a clinically important subgroup of sepsis because they are frequently associated with rapid hemodynamic deterioration, septic shock, antimicrobial resistance, and delays in the administration of active antimicrobial treatment. In addition, the availability of organism identification and antimicrobial susceptibility results often influences treatment decisions during the first several days of care. These characteristics may make reassessment at 72 h particularly relevant in this population, as the patient’s early clinical trajectory and response to antimicrobial and supportive treatment become more apparent. Nevertheless, the present study did not directly evaluate antimicrobial appropriateness, resistance mechanisms, or source control, and therefore did not aim to establish a pathogen-specific causal mechanism.

The 72 h time point during ICU stay represents a clinically relevant reassessment window. By this stage, early treatment response can be evaluated, organ-support strategies can be reconsidered, and microbiological identification and susceptibility results are often available. Therefore, clinical and laboratory measurements obtained at 72 h may provide prognostic information beyond that available at ICU admission, as they reflect both initial disease severity and the subsequent clinical course [9,10]. However, because alternative reassessment time points such as 24 or 48 h were not directly compared in the present study, 72 h should be regarded as a clinically pragmatic landmark rather than a uniquely optimal time point.

In addition to laboratory biomarkers, several clinical factors, including septic shock, organ dysfunction, vasopressor requirement, and the need for mechanical ventilation, have been associated with mortality in patients with sepsis. Their prognostic interpretation, however, may be influenced by disease severity, treatment exposure, and time-dependent selection effects [11,12]. This consideration is particularly important in analyses restricted to patients who remain alive at a later reassessment time point.

Despite growing interest in serial assessment, few studies have directly compared baseline and 72 h multivariable prognostic models in patients with Gram-negative sepsis while explicitly accounting for the landmark nature of the analysis. Therefore, this study aimed to evaluate the prognostic value of baseline and 72 h clinical, laboratory, and severity-score parameters for 28-day mortality in a 72 h landmark cohort of ICU patients with Gram-negative bloodstream infections and to determine whether the 72 h model provided greater prognostic discrimination than the baseline model.

## 2. Materials and Methods

### 2.1. Study Design and Population

This retrospective single-center cohort study was conducted at Mersin City Training and Research Hospital between 1 January 2018 and 1 November 2023. The medical records of adult patients admitted to the ICU with sepsis or septic shock were reviewed. Baseline was defined as the time of ICU admission for a sepsis episode meeting Sepsis-3 criteria. Clinical and laboratory measurements obtained during the initial ICU evaluation were considered baseline values.

Patients were eligible if they were aged 18 years or older, fulfilled the Sepsis-3 diagnostic criteria, and had at least one blood culture obtained during the initial sepsis evaluation that subsequently yielded a Gram-negative bacterial pathogen. Patients with bloodstream infections that developed later during ICU follow-up were not included. All patients received empirical antimicrobial therapy at the time of sepsis recognition. Antimicrobial treatment was subsequently reviewed according to microbiological identification, antimicrobial susceptibility results, and infectious diseases consultation when available.

### 2.2. Exclusion Criteria and Cohort Formation

Patients with incomplete clinical or laboratory data and those admitted to the ICU primarily for trauma-related conditions or postoperative care were excluded. Patients who died before the 72 h assessment were also excluded because 72 h clinical and laboratory measurements could not be obtained. Accordingly, the primary analysis was conducted as a 72 h landmark cohort analysis with a fixed-horizon binary outcome, and its findings apply only to patients who survived to this landmark.

**Figure 1:**
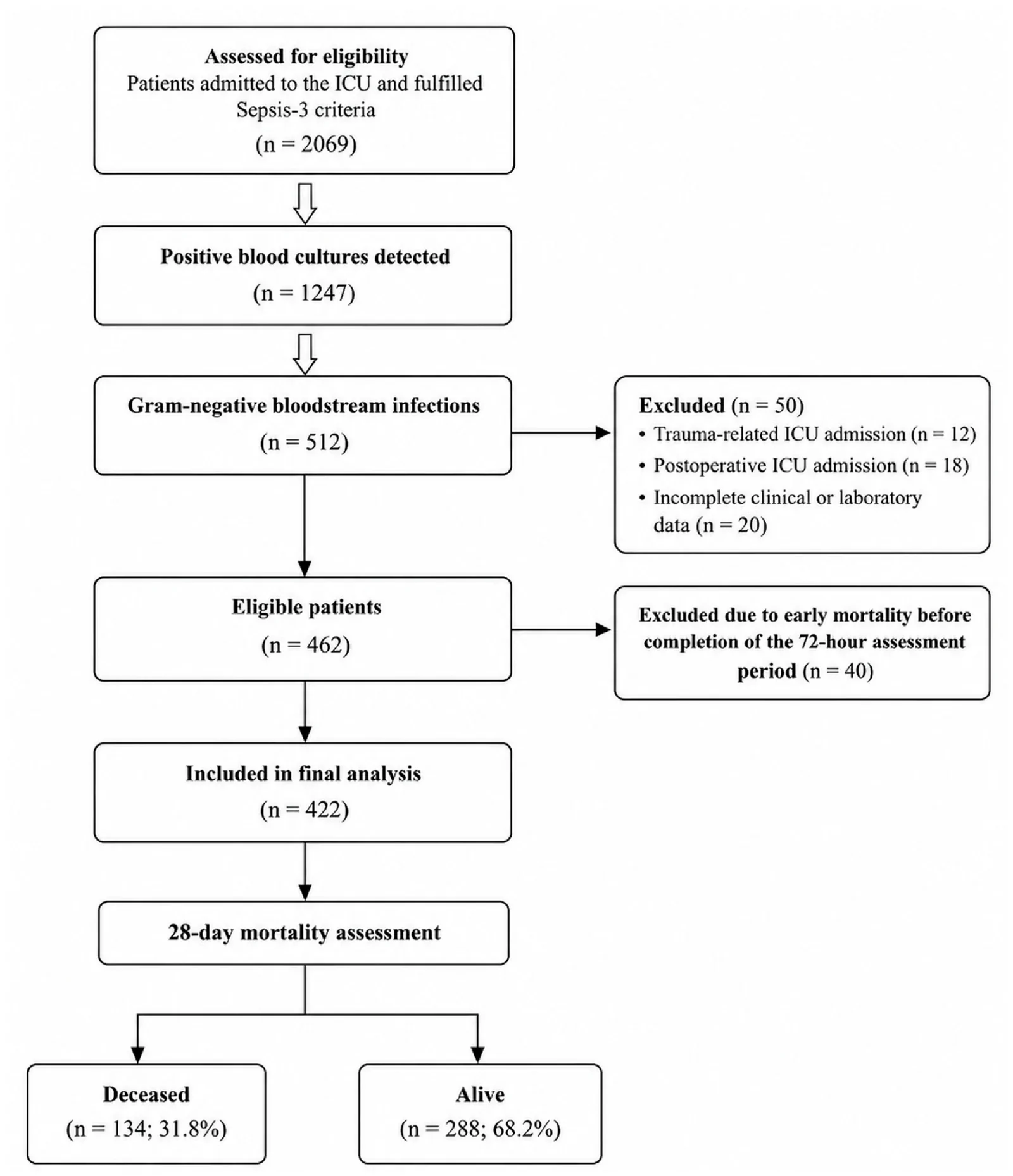
Flow diagram of patient selection from ICU admissions fulfilling Sepsis-3 criteria to the final study cohort of patients with Gram-negative bloodstream infections.

During the study period, 2069 patients admitted to the ICU fulfilled the Sepsis-3 criteria. Of these, 1247 had positive blood cultures, and 512 had Gram-negative bloodstream infections. Patients admitted for trauma-related conditions (n = 12), postoperative care (n = 18), or with incomplete clinical or laboratory data (n = 20) were excluded, leaving 462 eligible patients. A further 40 patients who died before completion of the 72 h assessment were excluded from the landmark cohort. Ultimately, 422 patients were included in the final analysis (Figure 1).

Analyses were performed using complete cases, and no imputation was applied.

### 2.3. Data Collection

Clinical and demographic data extracted from the medical records included age, sex, presence of baseline septic shock, vasopressor therapy within the first 24 h, use of invasive mechanical ventilation, and the timing of mechanical ventilation initiation during the first 72 h. Disease severity was assessed using the Sequential Organ Failure Assessment (SOFA) and Acute Physiology and Chronic Health Evaluation II (APACHE II) scores. The 72 h mark APACHE II score included core temperature, mean arterial pressure, heart rate, respiratory rate, oxygenation (PaO2 or A-a gradient), arterial pH, serum sodium, potassium, creatinine, hematocrit, leukocyte count, and the Glasgow Coma Scale score. These values were used to compute the Acute Physiology Score (APS). The APS was subsequently combined with age-weighted points (applied to patients >44 years) and chronic health points. Chronic health points were assigned upon review of the patient’s medical history for severe, pre-existing organ insufficiency (hepatic, cardiovascular, renal, or respiratory) or severe immunosuppression, with the specific weighting dependent on whether the ICU admission was categorized as elective operative emergency/non-operative. The final composite 72 h mark APACHE II score was recorded as a continuous variable for statistical analysis. Laboratory and physiological variables included complete blood count parameters, C-reactive protein (CRP), procalcitonin (PCT), and arterial blood gas measurements, including partial pressure of oxygen, partial pressure of carbon dioxide, and lactate. Blood culture results and the identified Gram-negative microorganisms were also recorded.

SOFA score, APACHE II score, PCT, CRP, lactate, and neutrophil-to-lymphocyte ratio (NLR) were recorded at baseline and at 72 h. Lactate clearance was also calculated. Delta values were defined as the absolute difference between the 72 h and baseline measurements:

Δ = value at 72 h − baseline value.

Accordingly, a positive delta value indicated an increase, whereas a negative delta value indicated a decrease during the first 72 h.

Among patients who were alive and under observation at the 72 h landmark, the primary outcome was all-cause death occurring after the 72 h landmark and before day 28 following ICU admission.

### 2.4. Definitions

Sepsis was defined according to the Sepsis-3 criteria as life-threatening organ dysfunction caused by a dysregulated host response to infection, operationalized as an acute increase of at least 2 points in the Sequential Organ Failure Assessment (SOFA) score. Septic shock was defined as sepsis requiring vasopressor therapy to maintain a mean arterial pressure of at least 65 mmHg despite adequate fluid resuscitation, together with a serum lactate concentration greater than 2 mmol/L [13,14]. For all analyses, septic shock status was assessed at baseline, defined as the time of ICU admission for the qualifying sepsis episode.

Gram-negative bloodstream infection was defined as the isolation of at least one Gram-negative bacterial pathogen from a blood culture obtained during the initial evaluation of the sepsis episode leading to ICU admission. For patients with more than one episode of Gram-negative bloodstream infection during the study period, only the first eligible episode was included in the analysis. *Escherichia coli, Pseudomonas* spp., and *Klebsiella* spp. were the only Gram-negative organisms identified among patients who met the predefined eligibility criteria and were included in the final 72 h landmark cohort; therefore, no additional Gram-negative organisms were available for analysis.

Mechanical ventilation was defined as the use of invasive mechanical ventilatory support during ICU follow-up. Initiation within the first 24 h was recorded as a separate timing variable. Mechanical ventilation duration was categorized as none, ≤24 h, >24–72 h, and >72 h.

The neutrophil-to-lymphocyte ratio (NLR) was calculated by dividing the absolute neutrophil count by the absolute lymphocyte count. Lactate clearance was calculated using the following formula:

Lactate clearance (%) = [(baseline lactate − 72 h lactate)/baseline lactate] × 100.

A positive lactate clearance value indicated a reduction in lactate concentration over the first 72 h, whereas a zero or negative value indicated no reduction or an increase in lactate concentration [15].

### 2.5. Statistical Analysis

Statistical analyses were performed using IBM SPSS Statistics version 23.0 (IBM Corp., Armonk, NY, USA). DeLong’s test and optimism-corrected internal validation and calibration analyses were performed using Python version 3.13.5 with the statsmodels and scikit-learn packages. The distribution of continuous variables was assessed using the Kolmogorov–Smirnov or Shapiro–Wilk test, as appropriate. Normally distributed continuous variables were presented as mean ± standard deviation, whereas non-normally distributed variables were presented as median and interquartile range. Categorical variables were expressed as counts and percentages.

Comparisons between 28-day survivors and non-survivors were performed using the independent-samples Student’s t-test or Mann–Whitney U test for continuous variables and the Pearson chi-square test or Fisher’s exact test for categorical variables, as appropriate. Exploratory associations among selected continuous, ordinal, and binary variables were evaluated using Spearman’s rank correlation coefficient. All statistical tests were two-sided, and a *p*-value < 0.05 was considered statistically significant.

Because the principal objective was to evaluate prognostic information available at 72 h, the primary analyses were conducted as a 72 h landmark cohort analysis with a fixed-horizon binary outcome. The analysis was restricted to patients who were alive and under observation at 72 h and had complete clinical and laboratory measurements at that time point. This approach differs from a classical landmark time-to-event survival analysis because logistic regression was used to evaluate death occurring between the 72 h landmark and day 28. Mortality during this post-landmark period was coded as the event outcome (1 = death; 0 = survival).

Separate multivariable logistic regression models were constructed using baseline and 72 h variables to evaluate their associations with mortality occurring between the 72 h landmark and day 28. Although serum lactate is included in the Sepsis-3 definition of septic shock, baseline lactate was retained as a continuous variable because it captures the magnitude of metabolic derangement beyond the binary classification of baseline septic shock. Their simultaneous inclusion was considered clinically justified because baseline septic shock represents a composite binary clinical syndrome, whereas baseline lactate provides continuous information on metabolic severity. Candidate predictors were selected based on clinical relevance, previously reported associations with sepsis mortality, and findings from exploratory univariable analyses.

Multicollinearity among predictors was assessed using tolerance, variance inflation factor, and condition index values. A tolerance value <0.20, a variance inflation factor >5, or a condition index >30 was considered indicative of potentially relevant multicollinearity.

Model fit was evaluated using the omnibus likelihood-ratio test, −2 log likelihood, Cox–Snell R^2^, Nagelkerke R^2^, and the Hosmer–Lemeshow goodness-of-fit test. Discrimination was assessed using receiver operating characteristic curve analysis and the area under the receiver operating characteristic curve (AUC), with 95% confidence intervals. Classification performance was evaluated at a predicted-probability threshold of 0.50, and sensitivity, specificity, and overall classification accuracy were calculated.

The AUCs of the baseline and 72 h models were compared using DeLong’s test for correlated ROC curves. Internal validation of the final 72 h model was performed using an optimism-correction bootstrap procedure with 1000 resamples. In each resample, the complete model was refitted in the bootstrap sample and evaluated in both the bootstrap sample and the original cohort. Optimism was estimated as the difference between model performance in the bootstrap sample and its performance when applied to the original cohort. Optimism-corrected AUC and Brier score were subsequently calculated. Calibration was assessed using the optimism-corrected calibration intercept and slope, with ideal values of 0 and 1, respectively, and was additionally examined using a calibration plot comparing observed and predicted 28-day mortality across deciles of predicted risk.

## 3. Results

A total of 422 patients were included in the 72 h landmark cohort. The median age was 69 years (interquartile range, 58–77), and 160 patients (37.9%) were female. Overall, 134 patients (31.8%) died within 28 days, whereas 288 (68.2%) survived. Descriptive statistics for continuous and categorical variables are presented in Supplementary Tables S1 and S2, respectively.

Patients were stratified according to mortality occurring between the 72 h landmark and day 28 status. Compared with survivors, non-survivors had significantly higher baseline and 72 h SOFA and APACHE II scores (all *p* < 0.001). At 72 h, neutrophil count, NLR, PCT, CRP, ALT, creatinine, and lactate levels were also significantly higher among non-survivors (*p* = 0.006, *p* = 0.006, *p* = 0.008, *p* < 0.001, *p* = 0.029, *p* = 0.003, and *p* < 0.001, respectively). Changes in PCT and lactate over the first 72 h differed significantly between the groups, with non-survivors showing smaller reductions in both markers (ΔPCT, *p* = 0.013; Δlactate, *p* = 0.006). Lactate clearance was significantly lower among non-survivors than survivors (*p* < 0.001) (Table 1).

Baseline septic shock and vasopressor therapy were significantly more frequent among non-survivors than survivors (*p* < 0.001 and *p* = 0.004, respectively). Invasive mechanical ventilation also differed significantly between the groups (*p* < 0.001); however, its inverse association with mortality should be interpreted cautiously in the context of selection at the 72 h landmark. Detailed categorical comparisons are presented in Table 2.

Spearman’s correlation analysis showed a strong positive association between baseline septic shock and initiation of vasopressor therapy within the first 24 h (r_s = 0.745, *p* < 0.001). A strong positive correlation was also observed between 72 h PCT and CRP levels (r_s = 0.718, *p* < 0.001). In addition, 72 h PCT was moderately correlated with 72 h creatinine (r_s = 0.315, *p* < 0.001), initiation of invasive mechanical ventilation (r_s = 0.303, *p* < 0.001), and duration of invasive mechanical ventilation (r_s = 0.303, *p* < 0.001). Baseline APACHE II scores were moderately correlated with 72 h APACHE II scores (r_s = 0.480, *p* < 0.001). A weaker but statistically significant positive correlation was observed between baseline and 72 h SOFA scores (r_s = 0.349, *p* < 0.001).

In univariable logistic regression analyses, baseline septic shock, vasopressor therapy, invasive mechanical ventilation, baseline and 72 h SOFA scores, baseline and 72 h APACHE II scores, 72 h CRP, 72 h PCT, 72 h lactate, ΔPCT, and Δlactate were significantly associated with post-landmark mortality through day 28. The inverse association observed for invasive mechanical ventilation was interpreted cautiously because the analysis was restricted to patients who survived to the 72 h landmark. The complete univariable results and the units or reference categories used for each predictor are presented in Supplementary Table S5. The candidate predictors considered for the 72 h multivariable model and the rationale for their inclusion or exclusion are summarized in Supplementary Table S4.

No evidence of relevant multicollinearity was identified among the predictors included in the final 72 h model. Tolerance values ranged from 0.876 to 0.961, variance inflation factor values ranged from 1.040 to 1.141, and the maximum condition index was 6.725, all of which were below the prespecified thresholds for potentially relevant multicollinearity (Supplementary Table S3).

In the final multivariable logistic regression model, baseline septic shock, 72 h SOFA score, 72 h CRP, and 72 h lactate were independently associated with post-landmark mortality through day 28. Baseline septic shock was associated with approximately fourfold higher odds of mortality (adjusted OR = 3.994; 95% CI: 2.296–6.949; *p* < 0.001). Each 1-point increase in the 72 h SOFA score was associated with a 60.3% increase in the odds of mortality (adjusted OR = 1.603; 95% CI: 1.424–1.805; *p* < 0.001). Higher 72 h CRP (adjusted OR = 1.005 per 1 mg/L increase; 95% CI: 1.001–1.008; *p* = 0.005) and higher 72 h lactate (adjusted OR = 1.189 per 1 mmol/L increase; 95% CI: 1.007–1.403; *p* = 0.041) were also independently associated with mortality (Table 3).

The final model was statistically significant (omnibus χ^2^ = 158.882, df = 4, *p* < 0.001), with a Nagelkerke R^2^ of 0.440. The Hosmer–Lemeshow test showed no evidence of poor fit (χ^2^ = 9.973, df = 8, *p* = 0.267). The model demonstrated good discrimination, with an apparent AUC of 0.867 (95% CI: 0.831–0.902). At a predicted-probability threshold of 0.50, the model correctly classified 78.7% of patients, with a sensitivity of 56.0% and a specificity of 89.2% for post-landmark mortality through day 28.

**Figure 2:**
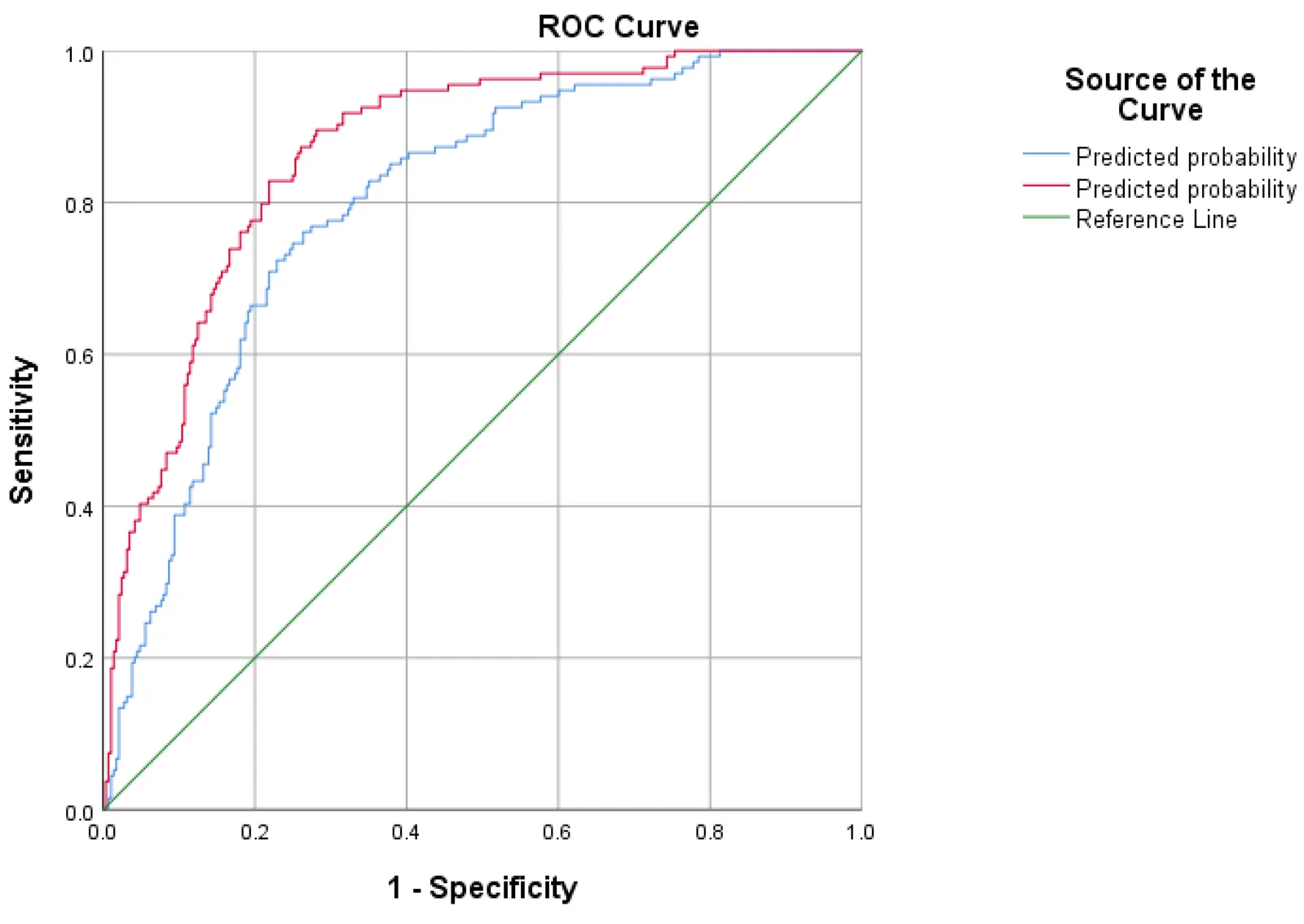
Receiver operating characteristic curves of the baseline and 72 h models for predicting mortality occurring between the 72 h landmark and day 28 in the 72 h landmark cohort (n = 422). The baseline model included baseline septic shock, baseline SOFA score, baseline CRP, and baseline lactate, whereas the 72 h model included baseline septic shock, 72 h SOFA score, 72 h CRP, and 72 h lactate. The baseline model demonstrated an AUC of 0.799 (95% CI: 0.755–0.842), whereas the 72 h model demonstrated an AUC of 0.867 (95% CI: 0.831–0.902). The 72 h model showed significantly greater discrimination than the baseline model, with an AUC difference of 0.068 (95% CI: 0.027–0.109; DeLong z = 3.232; *p* = 0.0012).

The baseline model, which included baseline septic shock, baseline SOFA score, baseline CRP, and baseline lactate, demonstrated an AUC of 0.799 (95% CI: 0.755–0.842), a Nagelkerke R^2^ of 0.303, and an overall classification accuracy of 73.5%. The Hosmer–Lemeshow test showed no evidence of poor fit for the baseline model (*p* = 0.192). In comparison, the 72 h model demonstrated an AUC of 0.867 (95% CI: 0.831–0.902), a Nagelkerke R^2^ of 0.440, and an overall classification accuracy of 78.7%. Paired comparison of the correlated ROC curves using DeLong’s test showed that the 72 h model had significantly greater discrimination than the baseline model (AUC difference = 0.068; 95% CI: 0.027–0.109; z = 3.232; *p* = 0.0012) (Table 4). Receiver operating characteristic curves for both models are presented in Figure 2.

**Figure 3:**
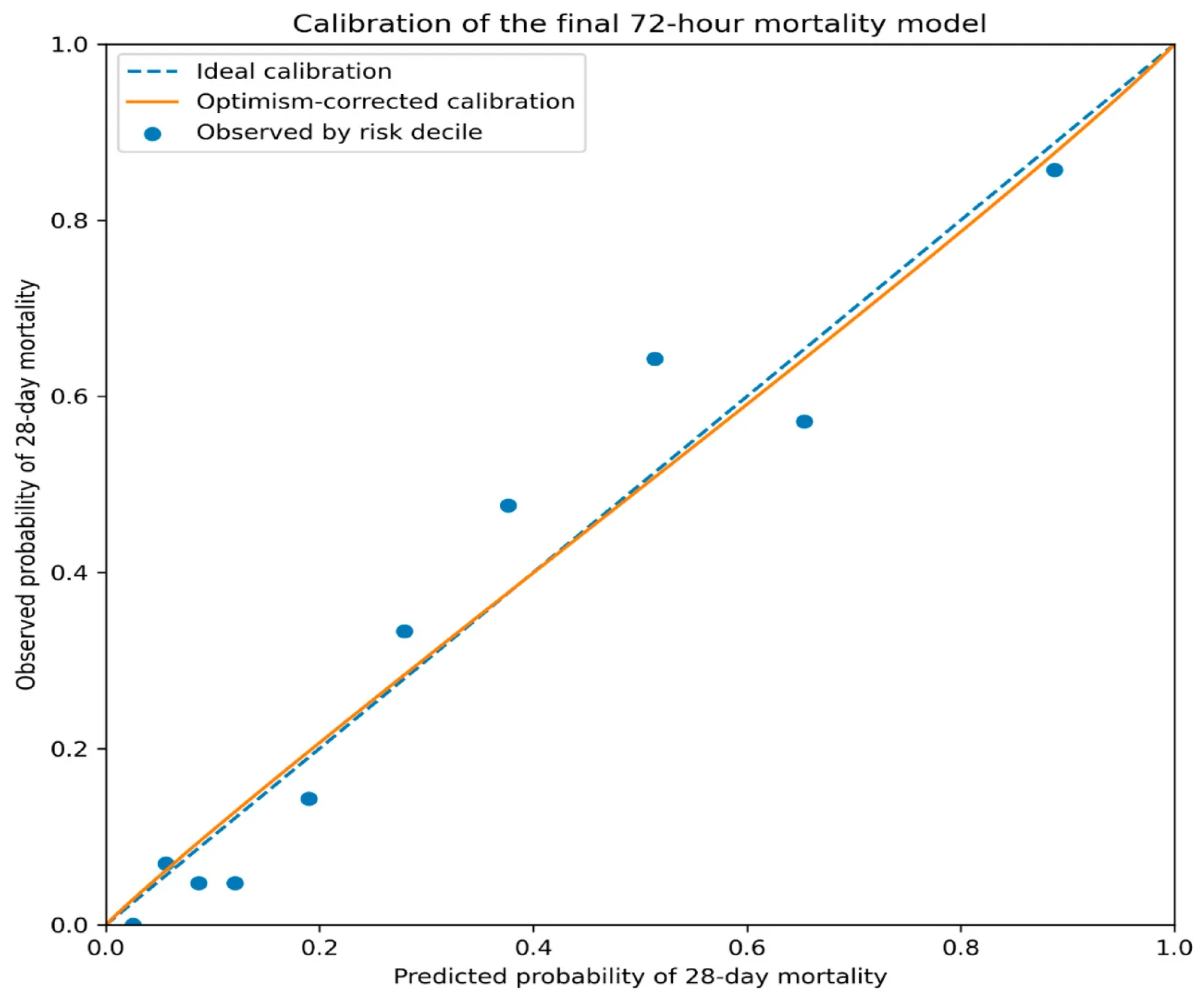
Calibration plot of the final 72 h model for predicting 28-day mortality in the 72 h landmark cohort (n = 422). The dashed diagonal line represents perfect calibration. Points indicate observed mortality across deciles of predicted risk, and the calibration curve represents optimism-corrected model calibration based on 1000 bootstrap resamples. The optimism-corrected calibration intercept was −0.020, the calibration slope was 0.956, and the optimism-corrected Brier score was 0.143.

Internal validation using 1000 bootstrap resamples yielded a mean optimism of 0.006 and an optimism-corrected AUC of 0.861. The optimism-corrected Brier score was 0.143, with a calibration intercept of −0.020 and a calibration slope of 0.956. The calibration plot showed generally close agreement between predicted and observed mortality risk (Figure 3).

## 4. Discussion

This retrospective single-center study evaluated the prognostic value of 72 h clinical and laboratory reassessment in ICU patients with sepsis and Gram-negative bloodstream infections. In the 72 h landmark cohort of 422 patients, the model based on reassessment at 72 h demonstrated significantly greater discrimination for mortality occurring between the 72 h landmark and day 28 than the baseline model. Baseline septic shock, 72 h SOFA score, 72 h CRP, and 72 h lactate were independently associated with mortality. The 72 h model achieved an apparent AUC of 0.867, compared with 0.799 for the baseline model, and this difference was statistically significant. Internal validation yielded an optimism-corrected AUC of 0.861, with a calibration slope of 0.956 and a Brier score of 0.143. The 72 h model demonstrated significantly greater discrimination than the baseline model for mortality occurring between the 72 h landmark and day 28. Sepsis is a dynamic and rapidly evolving syndrome, and measurements obtained at ICU admission primarily reflect the initial severity of illness. In contrast, reassessment at 72 h may capture persistent organ dysfunction, metabolic abnormalities, and the early clinical response to treatment. In the present study, baseline septic shock was independently associated with mortality occurring between the 72 h landmark and day 28, with approximately fourfold higher odds of death among patients with septic shock than among those without shock (adjusted OR = 3.994; 95% CI: 2.296–6.949). This finding is consistent with the Sepsis-3 framework, in which septic shock represents a subgroup of sepsis characterized by profound circulatory and metabolic abnormalities and a higher risk of mortality [16]. It is also in agreement with previous studies of Gram-negative severe sepsis and septic shock showing that hemodynamic instability and shock are strongly associated with adverse outcomes [17].

In the present study, both Δlactate and lactate clearance differed significantly between survivors and non-survivors, suggesting that the early trajectory of lactate may provide prognostic information beyond a single admission measurement. Previous work has similarly shown that dynamic lactate indices, including the magnitude and rate of lactate change, may reflect ongoing physiological derangement more accurately than an isolated baseline value [18]. Consistent with this concept, 72 h lactate was independently associated with 28-day mortality in the final multivariable model, whereas baseline lactate was not significantly associated with mortality in the univariable analysis. These findings support the prognostic relevance of persistent lactate elevation at 72 h; however, they do not establish that lactate clearance itself is an independent predictor because clearance was not included in the final multivariable model.

The SOFA score quantifies the extent of organ dysfunction across multiple physiological systems and is particularly suitable for serial assessment during critical illness. Rather than serving solely as a static mortality prediction tool, repeated SOFA measurements can provide information regarding the evolution of organ dysfunction over time [19]. Previous studies have demonstrated that worsening or persistently elevated SOFA scores during the early ICU course are associated with adverse outcomes. Ferreira et al. reported that, irrespective of the initial score, an increase in SOFA score during the first 48 h of ICU admission was associated with a mortality rate of at least 50% [20]. Similarly, Jones et al. evaluated SOFA scores at initial presentation and 72 h after ICU admission in patients with severe sepsis and found that the 72 h assessment provided clinically relevant prognostic information [21]. Consistent with these observations, the 72 h SOFA score was independently associated with 28-day mortality in our landmark cohort, with each 1-point increase corresponding to a 60.3% increase in the odds of death. These findings indicate that the severity of organ dysfunction persisting at 72 h may be more informative than admission measurements alone among patients who survive to this reassessment point.

Our findings also support the potential prognostic value of CRP measured at 72 h. Although both CRP and PCT are widely used for monitoring inflammatory activity in sepsis, only 72 h CRP was retained in the final multivariable model, where higher levels were independently associated with 28-day mortality. Previous studies have similarly reported associations between persistently elevated CRP concentrations or limited CRP decline and adverse outcomes in critically ill patients with sepsis [22,23]. In the present cohort, 72 h PCT and CRP levels were strongly positively correlated (r_s = 0.718, *p* < 0.001), indicating that the two biomarkers provided related, although not identical, information regarding inflammatory activity. However, this correlation should not be interpreted as evidence that either marker directly reflects a specific pathogen-related inflammatory mechanism. Although 72 h PCT was associated with mortality in univariable analysis, it was not retained in the final model, whereas 72 h CRP remained independently associated with mortality. Therefore, the present findings suggest that CRP contributed additional prognostic information within this parsimonious multivariable model, rather than demonstrating that CRP is universally superior to PCT.

In univariable analyses, invasive mechanical ventilation was inversely associated with 28-day mortality. This unexpected finding should be interpreted cautiously because the analysis was restricted to patients who survived to the 72 h landmark. Exclusion of patients who died before 72 h may have introduced a time-dependent selection effect, whereby patients who survived long enough to receive or continue invasive mechanical ventilation were more likely to be represented in the landmark cohort. Therefore, this association should not be interpreted as evidence of a protective effect of mechanical ventilation [24]. Beyond this finding, the final 72 h model combined markers of organ dysfunction, systemic inflammation, metabolic impairment, and hemodynamic instability through the inclusion of SOFA score, CRP, lactate, and septic shock. This integrated approach may provide a broader representation of clinical severity than reliance on a single biomarker or severity score alone. However, its clinical utility requires confirmation through external validation before implementation in routine practice.

This study has several limitations. First, its retrospective observational design precludes causal inference and allows only the identification of associations between the evaluated variables and mortality. Second, the study was conducted at a single center, which may limit generalizability to institutions with different ICU admission practices, patient populations, and local antimicrobial resistance patterns. Third, the analysis was restricted to patients with Gram-negative bloodstream infections; therefore, the findings should not be extrapolated to Gram-positive or fungal sepsis. Fourth, detailed comorbidity burden was not incorporated into the prognostic models, and residual confounding cannot be excluded. In addition, antimicrobial appropriateness and timing, resistance mechanisms, infection source, source-control interventions, and other treatment-related factors were not evaluated and may have influenced outcomes. Fifth, patients who died before the 72 h assessment were excluded by design. Consequently, the findings apply only to patients who survived to the 72 h landmark and may be affected by time-dependent selection, limiting their applicability to patients with very early mortality. Sixth, reassessment at 72 h was not directly compared with alternative time points such as 24 or 48 h; therefore, the present results do not establish 72 h as the optimal reassessment interval. Finally, although bootstrap internal validation showed limited optimism and generally favorable calibration, the model has not undergone external validation and should not be implemented in routine clinical practice before confirmation in independent cohorts.

## 5. Conclusions

Among ICU patients with Gram-negative sepsis who were alive at the 72 h landmark, baseline septic shock, 72 h SOFA score, 72 h CRP, and 72 h lactate were independently associated with subsequent mortality through day 28. The 72 h model demonstrated significantly greater discrimination than the baseline model and showed limited optimism after internal bootstrap validation. External validation is required before routine clinical use.

## Figures and Tables

**Table 1:** Comparison of continuous clinical and laboratory variables between 28-day survivors and non-survivors in the 72 h landmark cohort (n = 422).

Variable	Outcome	n	Median	IQR	U	*p*
Age, years	Non-survivors	134	67	17.25	17,087.0	0.058
	Survivors	288	70	21		
Baseline SOFA score	Non-survivors	134	9	3.25	9976.0	<0.001
	Survivors	288	6	4		
Baseline APACHE II score	Non-survivors	134	16	10	13,725.0	<0.001
	Survivors	288	12	7		
Baseline WBC, cells/µL	Non-survivors	134	12,200	9312.5	19,086.5	0.857
	Survivors	288	11,525	9762.5		
Baseline platelet count, ×10^3^/µL	Non-survivors	134	207	171.5	19,107.0	0.871
	Survivors	288	206.5	136.75		
Baseline lymphocyte count, cells/µL	Non-survivors	134	805	1270	18,262.5	0.376
	Survivors	288	850	1180		
Baseline neutrophil count, cells/µL	Non-survivors	134	10,150	9800	19,274.0	0.985
	Survivors	288	8940	9300		
Baseline NLR	Non-survivors	134	10.531	16.91	18,749.5	0.639
	Survivors	288	9.882	15.64		
Baseline PCT, ng/mL	Non-survivors	134	9.35	28.18	18,275.5	0.381
	Survivors	288	7.71	38.68		
Baseline CRP, mg/L	Non-survivors	134	64	142.15	18,731.0	0.628
	Survivors	288	46	123		
Baseline ALT, U/L	Non-survivors	134	21.5	28.25	19,045.0	0.829
	Survivors	288	23	29		
Baseline creatinine, mg/dL	Non-survivors	134	1.32	1.81	19,236.5	0.959
	Survivors	288	1.30	1.47		
Baseline lactate, mmol/L	Non-survivors	134	2.80	2.41	18,836.0	0.693
	Survivors	288	2.70	1.84		
72 h SOFA score	Non-survivors	134	8.0	2.0	7142.0	<0.001
	Survivors	288	6.0	2.25		
72 h APACHE II score	Non-survivors	134	13	10.25	14,779.5	<0.001
	Survivors	288	12	8.0		
72 h WBC, cells/µL	Non-survivors	134	13,530	10,420	17,320.0	0.090
	Survivors	288	12,315	9535		
72 h platelet count, ×10^3^/µL	Non-survivors	134	200	184.75	17630.0	0.153
	Survivors	288	213.5	182.75		
72 h lymphocyte count, cells/µL	Non-survivors	134	790	500	17,602.5	0.146
	Survivors	288	820	710		
72 h neutrophil count, cells/µL	Non-survivors	134	10,950	10,447.5	16,063.0	0.006
	Survivors	288	8725	8832.5		
72 h NLR	Non-survivors	134	13.233	16.07	16,098.5	0.006
	Survivors	288	9.943	12.93		
72 h PCT, ng/mL	Non-survivors	134	0.480	14.86	16,198.5	0.008
	Survivors	288	0.385	4.01		
72 h CRP, mg/L	Non-survivors	134	46.5	140.8	14,599.0	<0.001
	Survivors	288	33.5	54.0		
72 h ALT, U/L	Non-survivors	134	50	66.25	16,747.0	0.029
	Survivors	288	35	42.5		
72 h creatinine, mg/dL	Non-survivors	134	2.20	1.63	15,787.5	0.003
	Survivors	288	2.00	1.50		
72 h lactate, mmol/L	Non-survivors	134	1.79	1.42	14,180.0	<0.001
	Survivors	288	1.40	0.92		
ΔSOFA	Non-survivors	134	−1	3	18,098.0	0.302
	Survivors	288	−2	6.00		
ΔAPACHE II	Non-survivors	134	−3	7.25	18,522.0	0.504
	Survivors	288	−3	6		
ΔPCT, ng/mL	Non-survivors	134	−3.975	15.15	16,410.5	0.013
	Survivors	288	−5.09	24.48		
ΔLactate, mmol/L	Non-survivors	134	−0.90	2.26	16,080.0	0.006
	Survivors	288	−1.30	1.87		
Lactate clearance, %	Non-survivors	134	33.33	68.97	14,443.5	<0.001
	Survivors	288	50.00	44.17		

Data are presented as median and interquartile range. Comparisons were performed using the Mann–Whitney U test. Δ was calculated as the 72 h value minus the baseline value; negative values indicate a decrease and positive values indicate an increase over the first 72 h. Abbreviations: IQR, interquartile range; U, Mann–Whitney U test statistic; SOFA, Sequential Organ Failure Assessment; APACHE II, Acute Physiology and Chronic Health Evaluation II; WBC, white blood cell count; NLR, neutrophil-to-lymphocyte ratio; PCT, procalcitonin; CRP, C-reactive protein; ALT, alanine aminotransferase. Units: WBC, neutrophil, and lymphocyte counts, cells/µL; platelet count, ×10^3^/µL; CRP, mg/L; PCT, ng/mL; lactate, mmol/L; creatinine, mg/dL; ALT, U/L; SOFA and APACHE II scores, points.

**Table 2:** Comparison of categorical characteristics according to mortality occurring between the 72 h landmark and day 28 in the 72 h landmark cohort (n = 422).

		Mortality					
		Non-Survivors		Survivors		*p*	Cramer’s V
		n	%	n	%		
Sex	Female	48	30.00	112	70.00	0.545	0.029
	Male	86	32.82	176	67.18		
Baseline septic shock	No	28	14.89	160	85.11	<0.001	0.325
	Yes	106	45.30	128	54.70		
Vasopressor therapy	No	44	24.31	137	75.69	0.004	0.139
	Yes	90	37.34	151	62.66		
Invasive mechanical ventilation	No	84	42.00	116	58.00	<0.001	0.209
	Yes	50	22.50	172	77.50		
Organisms	*E. coli*	60	30.00	140	70.00	0.680	0.043
	*Pseudomonas* spp.	38	34.86	71	65.14		
	*Klebsiella* spp.	36	31.86	77	68.14		

Data are presented as n (%). *p* values were calculated using the Pearson chi-square test. Cramér’s V was used to estimate effect size. Abbreviation: spp., species.

**Table 3:** Multivariable logistic regression analysis of factors associated with mortality occurring between the 72 h landmark and day 28 in the 72 h landmark cohort (n = 422).

Variable	Adjusted OR (95% CI)	B	SE	Wald	*p*
Baseline septic shock	3.994 (2.296–6.949)	1.385	0.282	24.033	<0.001
72 h SOFA score	1.603 (1.424–1.805)	0.472	0.060	60.863	<0.001
72 h CRP, mg/L	1.005 (1.001–1.008)	0.005	0.002	8.042	0.005
72 h lactate, mmol/L	1.189 (1.007–1.403)	0.173	0.084	4.188	0.041

Abbreviations: OR, odds ratio; CI, confidence interval; SE, standard error; CRP, C-reactive protein; SOFA, Sequential Organ Failure Assessment. Model note: Mortality occurring between the 72 h landmark and day 28 was coded as the event outcome (1 = death; 0 = survival); therefore, odds ratios greater than 1 indicate increased odds of mortality. The model included septic shock, 72 h SOFA score, 72 h CRP, and 72 h lactate, entered simultaneously. The overall model was statistically significant (omnibus χ^2^ = 158.882, df = 4, *p* < 0.001), with a −2 log likelihood of 368.616, Cox–Snell R^2^ of 0.314, and Nagelkerke R^2^ of 0.440. The Hosmer–Lemeshow test showed no evidence of poor fit (χ^2^ = 9.973, df = 8, *p* = 0.267). The apparent AUC was 0.867 (95% CI: 0.831–0.902). At a predicted-probability threshold of 0.50, the model correctly classified 78.7% of patients, with a sensitivity of 56.0% and a specificity of 89.2% for 28-day mortality. Internal validation using 1000 bootstrap resamples yielded a mean optimism of 0.006 and an optimism-corrected AUC of 0.861. The optimism-corrected calibration intercept was −0.020, the calibration slope was 0.956, and the optimism-corrected Brier score was 0.143.

**Table 4:** Comparison of baseline and 72 h models for predicting mortality occurring between the 72 h landmark and day 28.

Model	Predictors	AUC (95% CI)	Nagelkerke R^2^	Hosmer–Lemeshow *p*	Overall Accuracy
Baseline model	Baseline septic shock, baseline SOFA, baseline CRP, and baseline lactate	0.799 (0.755–0.842)	0.303	0.192	73.5%
72 h model	Baseline septic shock, 72 h SOFA, 72 h CRP, and 72 h lactate	0.867 (0.831–0.902)	0.440	0.267	78.7%

Abbreviations: AUC, area under the receiver operating characteristic curve; CI, confidence interval; CRP, C-reactive protein; SOFA, Sequential Organ Failure Assessment. Table note: Mortality occurring between the 72 h landmark and day 28 was coded as the event outcome (1 = death; 0 = survival). Classification accuracy was calculated using a predicted-probability threshold of 0.50. The Hosmer–Lemeshow test showed no evidence of poor fit for either model. Compared with the baseline model, the 72 h model had a higher AUC, greater Nagelkerke R^2^, and higher overall classification accuracy. The ROC curves were compared using DeLong’s test for correlated ROC curves. The 72 h model demonstrated significantly greater discrimination than the baseline model (AUC difference = 0.068; 95% CI: 0.027–0.109; z = 3.232; *p* = 0.0012).

## Data Availability

The data presented in this study are available on reasonable request from the corresponding author. The data are not publicly available due to institutional and patient privacy restrictions.

## Supplementary Materials

The following supporting information can be downloaded at: https://www.mdpi.com/article/10.3390/medicina62081531/s1. Supplementary Table S1: descriptive statistics of continuous variables in the 72 h landmark cohort; Supplementary Table S2: distribution of categorical variables in the 72 h landmark cohort; Supplementary Table S3: multicollinearity diagnostics for predictors included in the final 72 h model; Supplementary Table S4: candidate predictors considered for the 72 h multivariable mortality model and reasons for inclusion or exclusion; Supplementary Table S5: univariable logistic regression analysis of candidate predictors associated with 28-day mortality.
