# Supplementary material for: Prognostic Value of 72-h Reassessment Using SOFA Score, C-Reactive Protein, and Lactate in a 72-h Landmark Cohort of Intensive Care Patients with Gram-Negative Sepsis
Source: Medicina (Kaunas). 2026 Aug 9;62(8):1531. doi: 10.3390/medicina62081531 (PMC13515829; doi:10.3390/medicina62081531)
Supplement: Supplementary file 1 [file medicina-62-01531-s001.zip › medicina-4451849-supplementary.pdf]

Supplementary Table S1. Descriptive statistics of continuous variables in the 72-hour landmark cohort (n = 422).

Variable	Minimum	25th percentile	Median	75th percentile	Maximum
Age, years	18	58	69	77	98
Baseline SOFA score	1	6	8	9	17
Baseline APACHE II score	2	9	14	18	62
Baseline WBC, cells/µL	40	7938	11860	17523	209990
Baseline platelet count, ×10³/µL	8	140	208	292	4200
Baseline lymphocyte count, cells/µL	5	490	850	1700	53570
Baseline neutrophil count, cells/µL	0	5778	9530	15225	65940
Baseline NLR	0.00	4.50	10.21	20.20	639.50
Baseline PCT, ng/mL	0.10	2.00	8.38	34.85	128.00
Baseline CRP, mg/L	0.61	17.00	46.75	149.25	675.00
Baseline ALT, U/L	7.00	14.00	22.50	43.00	4520.00
Baseline creatinine, mg/dL	0.11	0.74	1.30	2.29	9.60
Baseline lactate, mmol/L	0.76	2.00	2.70	3.90	17.00
72-hour SOFA score	1	4	6	8	23
72-hour APACHE II score	2	8	12	18	42
72-hour WBC, cells/µL	80	8073	12600	17600	38270
72-hour platelet count, ×10³/µL	14	120	207	304	1070
72-hour lymphocyte count, cells/µL	2	510	800	1140	13670
72-hour neutrophil count, cells/µL	10	5100	9595	14925	35550
72-hour NLR	0.17	5.55	11.03	19.42	365.00
72-hour PCT, ng/mL	0.03	0.20	0.41	6.66	75.00
72-hour CRP, mg/L	2.00	20.00	39.00	100.00	570.00

72-hour ALT, U/L	6	22	38	75	2929
72-hour creatinine, mg/dL	0.20	1.40	2.00	2.90	31.00
72-hour lactate, mmol/L	0.16	1.02	1.51	2.16	16.00
ΔSOFA, points	−12	−3	−2	1	14
ΔAPACHE II, points	−56	−4	−3	2	30
ΔPCT, ng/mL	−122.00	−20.70	−4.52	−0.42	60.10
ΔLactate, mmol/L	−16.99	−2.32	−1.20	−0.40	14.67
Lactate clearance, %	−638.64	18.47	44.17	68.21	95.02

Abbreviations: ALT, alanine aminotransferase; APACHE II, Acute Physiology and Chronic Health Evaluation II; CRP, C-reactive protein; NLR, neutrophil-to-lymphocyte ratio; PCT, procalcitonin; SOFA, Sequential Organ Failure Assessment; WBC, white blood cell count; Δ, absolute difference calculated as the 72-hour value minus the baseline value.

Units: WBC, neutrophil, and lymphocyte counts, cells/µL; platelet count, $\times 10^3/\mu L$; CRP, mg/L; PCT, ng/mL; lactate, mmol/L; creatinine, mg/dL; and ALT, U/L.

Supplementary Table S2. Distribution of categorical variables in the 72-hour landmark cohort (n = 422).

Variable	Category	n	%
Sex	Female	160	37.9
	Male	262	62.1

Baseline septic shock	No	188	44.5
	Yes	234	55.5
Vasopressor therapy	No	181	42.9
	Yes	241	57.1
Invasive mechanical ventilation	No	200	47.4
	Yes	222	52.6
Duration of invasive mechanical ventilation, hours	None	200	47.4
	≤24	64	15.2
	>24–72	64	15.2
	>72	94	22.3
Mechanical ventilation initiated within the first 24 hours	No	358	84.8
	Yes	64	15.2
Bacterial species	*Escherichia coli*	200	47.4
	Pseudomonas spp.	109	25.8
	Klebsiella spp.	113	26.8
28-day outcome	Non-survivor	134	31.8
	Survivor	288	68.2

Data are presented as n (%). Percentages may not total 100% because of rounding. Mechanical ventilation duration represents the total duration of invasive mechanical ventilation. “Mechanical ventilation initiated within the first 24 hours” indicates initiation of invasive mechanical ventilation during the first 24 hours after ICU admission.

Abbreviations: ICU, intensive care unit; spp., species.

Supplementary Table S3. Multicollinearity diagnostics for predictors included in the final 72-hour model.

**Predictor**	**Tolerance**	**VIF**
Baseline septic shock	0.940	1.063
72-hour lactate, mmol/L	0.885	1.130
72-hour CRP, mg/L	0.876	1.141
72-hour SOFA score	0.961	1.040

Abbreviations: CRP, C-reactive protein; SOFA, Sequential Organ Failure Assessment; VIF, variance inflation factor.

Table note: Multicollinearity was assessed using tolerance, variance inflation factor, and condition index values. Tolerance < 0.20, VIF > 5, or a condition index > 30 was considered indicative of potentially relevant multicollinearity. Tolerance values ranged from 0.876 to 0.961, VIF values ranged from 1.040 to 1.141, and the maximum condition index was 6.725, indicating no evidence of relevant multicollinearity among the predictors included in the final model.

Supplementary Table S4. Candidate predictors considered for the 72-hour multivariable mortality model and reasons for inclusion or exclusion.

**Candidate predictor**	**Assessment time**	**Included in final model**	**Rationale**

Baseline septic shock	Baseline	Yes	Clinically established marker of sepsis severity and independently associated with 28-day mortality
SOFA score	72 hours	Yes	Represented persistent organ dysfunction at the landmark time point and was retained as the principal organ dysfunction measure
CRP	72 hours	Yes	Represented ongoing systemic inflammation and remained independently associated with mortality
Lactate	72 hours	Yes	Represented persistent metabolic dysfunction and remained independently associated with mortality
Baseline SOFA score	Baseline	No	Included in the separate baseline model; not entered simultaneously with 72-hour SOFA to avoid redundancy between measurements of the same construct
Baseline CRP	Baseline	No	Included in the separate baseline model; not entered simultaneously with 72-hour CRP
Baseline lactate	Baseline	No	Included in the separate baseline model; not entered simultaneously with 72-hour lactate
APACHE II score	Baseline/72 hours	No	Overlapped conceptually with SOFA as a global severity measure; SOFA was prioritized to maintain model parsimony and clinical interpretability
Procalcitonin	72 hours	No	Associated with mortality in univariable analysis; not retained to preserve model parsimony and avoid redundancy with other inflammatory and severity-related predictors
Vasopressor therapy	First 24 hours	No	Closely related to septic shock and therefore not entered simultaneously to avoid clinical redundancy
Mechanical ventilation	First 72 hours	No	Considered a clinically relevant severity marker; not retained because its interpretation was affected by 72-hour landmark selection and overlapped clinically with organ dysfunction severity

ΔSOFA	Baseline to 72 hours	No	Derived from baseline and 72-hour SOFA values and therefore not entered together with the 72-hour SOFA score
ΔCRP	Baseline to 72 hours	No	The originally derived values were affected by inconsistent CRP units before standardization; the standardized 72-hour CRP measurement was therefore used
ΔLactate	Baseline to 72 hours	No	Derived from baseline and 72-hour lactate values and provided overlapping information with the 72-hour lactate measurement
Lactate clearance	Baseline to 72 hours	No	Mathematically dependent on baseline and 72-hour lactate values and therefore not entered simultaneously with 72-hour lactate
NLR	Baseline/72 hours	No	Evaluated as an inflammatory index; not retained to maintain model parsimony because 72-hour CRP and established severity measures were prioritized
PLR	Baseline/72 hours	No	Excluded after source-data verification identified inconsistent platelet and lymphocyte measurement scales that prevented reliable recalculation

Abbreviations: APACHE II, Acute Physiology and Chronic Health Evaluation II; CRP, C-reactive protein; ICU, intensive care unit; NLR, neutrophil-to-lymphocyte ratio; PLR, platelet-to-lymphocyte ratio; SOFA, Sequential Organ Failure Assessment.

Table note: Candidate predictors were considered on the basis of clinical relevance, evidence from previous literature, and associations observed in univariable analyses. Baseline and 72-hour measurements of the same clinical construct, together with their derived delta or clearance variables, were not entered simultaneously into the same model to reduce redundancy and avoid overadjustment. The final 72-hour model included septic shock, 72-hour SOFA score, 72-hour CRP, and 72-hour lactate. All predictors were entered simultaneously using the enter method.

Supplementary Table S5. Univariable logistic regression analysis of candidate predictors associated with 28-day mortality in the 72-hour landmark cohort (n = 422).

Predictor	Unit/reference category	OR	95% CI	p
Age	Per 1-year increase	0.989	0.976–1.003	0.112
Male sex	Female as reference	1.140	0.745–1.744	0.545
Baseline septic shock	Yes vs. no	4.739	2.941–7.634	<0.001
Vasopressor therapy	Yes vs. no	1.856	1.209–2.848	0.005
Invasive mechanical ventilation	Yes vs. no	0.401	0.263–0.612	<0.001
Bacterial species	Overall comparison	—	—	0.681
Pseudomonas spp.	vs. *Escherichia coli*	1.249	0.760–2.052	0.381
Klebsiella spp.	vs. *Escherichia coli*	1.091	0.663–1.795	0.732
Baseline SOFA score	Per 1-point increase	1.302	1.209–1.402	<0.001
72-hour SOFA score	Per 1-point increase	1.602	1.432–1.791	<0.001
Baseline APACHE II score	Per 1-point increase	1.071	1.039–1.104	<0.001
72-hour APACHE II score	Per 1-point increase	1.061	1.031–1.092	<0.001
Baseline CRP	Per 1 mg/L increase	1.001	0.999–1.003	0.262
72-hour CRP	Per 1 mg/L increase	1.007	1.004–1.010	<0.001
Baseline PCT	Per 1 ng/mL increase	0.999	0.991–1.007	0.810
72-hour PCT	Per 1 ng/mL increase	1.019	1.008–1.031	0.001
Baseline lactate	Per 1 mmol/L increase	1.008	0.921–1.103	0.861

72-hour lactate	Per 1 mmol/L increase	1.361	1.158–1.600	<0.001
Baseline NLR	Per 1-unit increase	1.004	0.999–1.008	0.109
72-hour NLR	Per 1-unit increase	1.001	0.994–1.008	0.717
ΔSOFA	Per 1-point increase	1.026	0.968–1.087	0.382
ΔAPACHE II	Per 1-point increase	0.995	0.968–1.023	0.716
ΔPCT	Per 1 ng/mL increase	1.013	1.004–1.023	0.006
ΔLactate	Per 1 mmol/L increase	1.103	1.014–1.199	0.022
Lactate clearance	Per 1% increase	0.998	0.996–1.000	0.069

Abbreviations: APACHE II, Acute Physiology and Chronic Health Evaluation II; CI, confidence interval; CRP, C-reactive protein; NLR, neutrophil-to-lymphocyte ratio; OR, odds ratio; PCT, procalcitonin; SOFA, Sequential Organ Failure Assessment; spp., species; Δ, absolute difference calculated as the 72-hour value minus the baseline value.

Table note: Twenty-eight-day mortality was coded as the event outcome (1 = death; 0 = survival). Each predictor was evaluated in a separate univariable logistic regression model. Odds ratios for continuous variables represent the change in mortality odds per one-unit increase. *Escherichia coli* was used as the reference category for bacterial species. The inverse association observed for invasive mechanical ventilation should be interpreted cautiously because the analysis was restricted to patients who survived to the 72-hour landmark. ΔCRP and PLR were not included because source-data review identified unit inconsistencies that prevented reliable calculation.
